# Predicting Organ-Specific Risk Interactions between Radiation and Chemotherapy in Secondary Cancer Survivors

**DOI:** 10.3390/cancers9090119

**Published:** 2017-09-06

**Authors:** Venkata S.K. Manem, Clemens Grassberger, Harald Paganetti

**Affiliations:** Department of Radiation Oncology, Massachusetts General Hospital and Harvard Medical School, Boston, MA 02114, USA; mail2mvskumar@gmail.com (V.S.K.M.); Grassberger.Clemens@mgh.harvard.edu (C.G.)

**Keywords:** secondary malignancies, relative risks, chemotherapy-radiotherapy interaction, synergy

## Abstract

Several studies have shown that pediatric patients have an increased risk of developing a secondary malignancy several decades after treatment with radiotherapy and chemotherapy. In this work, we use a biologically motivated mathematical formalism to estimate the relative risks of breast, lung and thyroid cancers in childhood cancer survivors due to concurrent therapy regimen. This model specifically includes possible organ-specific interaction between radiotherapy and chemotherapy. The model predicts relative risks for developing secondary cancers after chemotherapy in breast, lung and thyroid tissues, and compared with the epidemiological data. For a concurrent therapy protocol, our model predicted relative risks of 3.2, 9.3, 4.5 as compared to the clinical data, i.e., 1.4, 8.0, 2.3 for secondary breast, lung and thyroid cancer risks, respectively. The extracted chemotherapy mutation induction rates for breast, lung and thyroid are 10^−9^, 0.5 × 10^−6^, 0.9 × 10^−7^ respectively. We found that there exists no synergistic interaction between radiation and chemotherapy for neither mutation induction nor cell kill in lung tissue, but there is an interaction in cell kill for the breast and thyroid organs. These findings help understand the risks of current clinical protocols and might provide rational guidance to develop future multi-modality treatment protocols to minimize secondary cancer risks.

## 1. Introduction

One of the success stories in the treatment of cancer in the past decades is the improvement in survival rates for childhood cancers: 5-year overall survival for all childhood cancers combined has increased from 58% in the late 1970s to 83% in the 2000s [1]. However, late toxicities remain a major concern in childhood cancer survivors. Several case control and epidemiological studies have suggested one of the causes of mortality in childhood cancer survivors are secondary neoplasms [2,3,4,5]. Most of the secondary tumors are found among survivors of Hodgkin’s lymphoma and sarcomas [6,7,8,9]. The two modes of treatments, radiation therapy as well as chemotherapy, can induce secondary cancer [10,11,12,13,14,15,16,17].

The first evidence of radiation-induced malignancies came from the atomic bomb survivor studies [10]. The impact of various radiotherapy techniques such as three-dimensional conformal radiotherapy (3DCRT), intensity modulated radiotherapy (IMRT) or proton therapy on secondary cancer induction is discussed in [11,12,13].

Epidemiological studies [14,15,16,17] also show the increased risk of chemotherapy agents to induce secondary cancers. This has been attributed to unrepaired or incorrectly repaired damage to healthy cells, causing mutations in following cells divisions [18]. Even if the induced mutation does not suffice for cancerous growth, the cell in question might acquire additional mutations resulting in a secondary neoplasm.

Most childhood cancers are currently treated with chemotherapeutic agents alone or in combination with radiotherapy. Our aim in this study is to develop a biologically motivated mathematical formalism to estimate the secondary cancer risks for lung, breast and thyroid tissue stemming from combined chemo-radiation treatments, and estimate the corresponding relative risks. All parameters used in this study are either taken from published studies or derived from published clinical data (see tables for source information). Furthermore, the model also includes the possibility of interaction between radiation and chemotherapeutic agents. The main objective is to establish a predictive model from available clinical data that can help design improved treatment schedules that lower the risk of secondary neoplasms in childhood cancer survivors.

## 2. Results

### 2.1. Radiation Alone

We estimate radiation-induced secondary cancer risks as excess relative risk (ERR), in order to be consistent with the results obtained by Sachs and Brenner [19]. We first extract the proliferation rates of cells in lung, breast and thyroid tissue from the historical data. The number of radiotherapy fractions typically administered, the cell kill and mutation rates for breast and lung are taken from [19]. The growth rate parameters (λ, *r*) in the system are extracted using radiation-induced secondary breast and lung cancer data by Sachs and Brenner (see [19]), and the proportionality factor, i.e., the function g  in Equation (1) for thyroid is assumed to be equal to one. The relative growth rate of pre-malignant thyroid cells was determined to be 0.68 by fitting the function to the historical ERR data [20]. Table 1 summarizes the parameters used to estimate radiation-induced carcinogenic risks.

**Figure 1:**
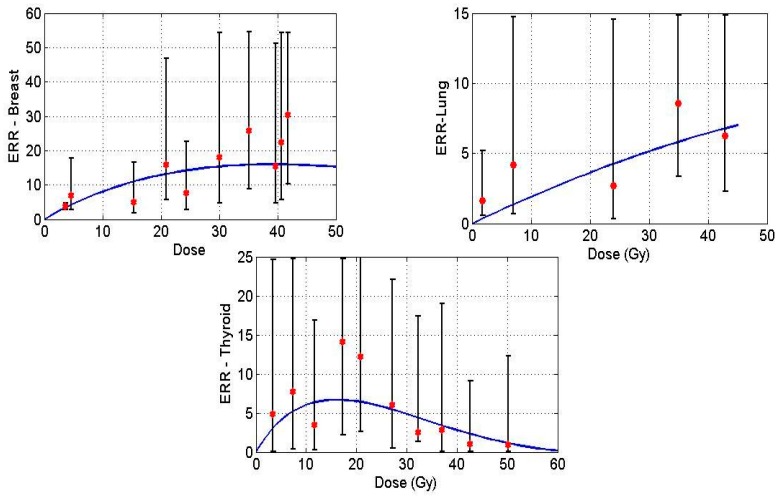
Excess relative risk (ERR) for breast (**upper left**), lung (**upper right**) and thyroid (**lower**) tissues plotted against dose. Data points taken from [20,22,23], solid curve is the mathematical model prediction.

Figure 1 displays model predictions with the historical breast, lung and thyroid data treated with radiation treatment only for Hodgkin’s Lymphoma (HL) patients. Solid lines in the figures denote the prediction made by the mathematical model, and the points indicate epidemiological data.

### 2.2. Chemotherapy Alone

We use the same model in the aforementioned section to describe the action of chemotherapy in normal tissue. We assume the same mechanisms to shape the dose-response curve for chemotherapy, i.e., cell growth, cell kill and mutation induction. For the case of “chemotherapy only”, we assume the growth rate of normal and pre-malignant cells and the total number of normal cells to be similar to that used for estimating radiation-induced cancer risks. Having extracted the relative growth rate of pre-malignant cells for thyroid in the previous section, we now investigate the case of secondary cancer induction through chemotherapy only. The parameters such as average number of days per chemotherapy cycle, number of cycles and dose per cycle are taken from the literature (see Table 2). Using the epidemiological data, we extract the mutation induction per cycle of chemotherapy for breast, lung and thyroid tissue. For this purpose, we assumed the cell kill in normal tissue to be 0.2 (m^2^/mg) per cycle, close to the radiotherapy only value, as there is no data for chemotherapy cell kill in normal tissue. The cell kill parameter should be interpreted as an average value of alkylating agents used in a specific regimen. In addition, we also performed a sensitivity analysis by altering the value of cell kill from 0.1 to 0.3, and examined its effect on the mutation induction rate. The order of magnitude of the mutation induction rate did not change with varying cell kill, meaning that our results are relatively robust towards our initial assumptions. We consider the total number of cells N = 10^6^ similar to the analysis on radiation-induced cancer risks. Table 2 summarizes chemotherapy related parameters that are taken from the literature. The number of chemotherapy cycles used in epidemiological studies [22,23] was quantified into three groups, 1–4, 5–8 and >9, however, in [20], groups were defined as lower, middle and upper tertiles. In order to approximate these groups quantitatively, we have chosen the average number of chemotherapy cycles for these groups to be 3, 6 and 10.

The proliferation rate of chemotherapy-induced pre-malignant cells is assumed to be the same as the growth rate of radiation-induced pre-malignant cells. Additionally, the proportional factor (g) used to estimate chemotherapy-induced risks is assumed to be the same as the one used to estimate radiation-induced carcinogenic risks. The model fit is carried out using secondary breast and lung cancer data in HL cancer survivors, and secondary thyroid cancer data from childhood cancer survivors [17,19,20].

To estimate a specific dosage of a specific drug is not possible, as a variety of chemotherapy drugs are used in different combinations in the treatment of childhood cancer. Therefore, we used the dosage of mechlorethamine (12 mg/m^2^) per cycle of MOPP (Mustargen, Oncovin, Procarbazine and Prednisone) [25], one of the most popular regimens during the time the data was taken [20,22,23], as substitute for the chemotherapy dose per cycle.

Following clinical practice, the fit was then carried out by varying the number of chemotherapy cycles $K_{C}$, while keeping the dosage per cycle fixed. The model fit for breast and lung is carried out by taking the average number of chemotherapy cycles per group (see, [20,22,23]) as 3, 6 and 10. From the model fit, we extracted the chemotherapy-induced mutation induction parameter per cycle for breast, lung and thyroid, which are presented in Table 3.

The low rate for chemotherapy-induced relative risks for breast is consistent with the historical data, which show that alkylating agents decrease the risk of secondary breast cancer in HL survivors [20]. Table 4 and Table 5 present a summary of relative risks predicted by a mathematical model against the epidemiological breast and lung data.

The overall increase or decrease in relative risk can also depend on the hormonal changes in the patient post treatment, especially in secondary breast cancer. Due to the scarcity of data with respect to hormonal status of a patient, we exclude the dependence of hormonal factors in this study. According to the formulation, ERR can asymptotically be close to zero but can never become zero, due to which we observe some relative risk for the breast organ.

As the low and medium exposed patients are grouped together in the data [17], we therefore take the average number of cycles to be 5, and 10 for the highly exposed group. Table 6 presents a summary of chemotherapy-induced relative risks for thyroid.

### 2.3. Concurrent Therapy

Many patients in the historical study have been given chemotherapeutic agents concurrently with radiation. In order to estimate the induced secondary cancer risks, it is essential to quantify the additional risk from the interaction between radiation and chemotherapy. In the following analysis, we want to explore if there is such an interaction and how it impacts secondary cancer risks.

All the concurrent therapy related parameters that we have derived from the clinical data up to this point will be used (see Table 1 and Table 2) to estimate the risk from concurrent therapy. All other parameters used are the same as the ones in Table 2 and Table 3.

Because the exact treatment schedules are not known, we assume that radiation is administered in the first cycle of chemotherapy. In the historical studies, radiation dose has been categorized into various groups (for instance, D = 0 Gy, D ≤ 20 Gy, D > 20 Gy) to quantify the associated secondary cancer risks. We assumed the average number of chemotherapy cycles to be 5 for all the three organs, estimated from the literature [20,22,23]. Furthermore, the average radiation dose for lung, breast and thyroid is taken to be 20 Gy, 25 Gy, 20 Gy respectively. Table 7 presents a summary of relative risks induced by concurrent therapy for all the critical organs, assuming that the terms for radio- and chemotherapy just act independently in terms of cell kill and mutation induction.

As can be seen from the model results in Table 7, the predicted relative risks for lung are close to the clinical values, well within the confidence intervals of the epidemiological data [23]. This indicates that radiation and chemotherapy might only have an additive effect for secondary cancer induction in these organs.

On the other hand, the model predicts the relative risks for breast and thyroid as ~9, ~7 respectively, far higher than those found in clinical studies [20,22]. As outlined in the methods, there is only one factor in the model that decreases the number of pre-malignant cells, and that is cell kill during therapy, because it can decrease the number of pre-malignant cells.

We therefore introduce an interaction term between the chemotherapeutic agent and radiation through a radiation sensitivity factor ε. This factor quantifies the radio-sensitization through chemotherapy within an organ of interest, and acts as a multiplier of the radiation dose. So we can fit the strength of the chemo-radiation interaction to the clinical data by adjusting the radiation sensitivity factor *ε*. Table 8 shows the results of the clinical data with an interacting term *ε* = 2.6, the relative risks now within the confidence intervals from the historical data.

Summing up, our model predicts relative risks (RR) for lung within the confidence intervals of the epidemiological data, while for thyroid and breast we introduced a radio-sensitization factor for cell kill to bring the model prediction in line with the clinical data [20,22]. This implies that chemo-radiation interactions are highly tissue dependent.

## 3. Discussion

The main novel aspect of this work is the fact that we treat secondary cancer induction by radiation or chemotherapy in the same biologically motivated mechanistic model, providing a unified framework for two treatment modalities that are often used, but rarely modeled, together. Mathematical model predictions of concurrent therapy-induced relative risks fit well within the error bounds of the epidemiological data. To summarize our results, we obtained the following:

- growth rate of radiation-induced pre-malignant cells in thyroid was obtained using a least squares fit to published data (which has been already performed in [19] for lung and breast)
- organ-specific chemotherapy-induced mutation rates were extracted using clinical data for breast, lung and thyroid, and varied by orders of magnitude between these organs
- the data for concurrent chemo-radiation indicates that there is no synergistic interaction between radiation and chemotherapy in lung tissue, however, in the case of breast and thyroid, there could be a sensitization effect for cell kill. The radio-sensitization factor we used (*ε* = 2.6) is in the range of values reported in the literature for mechlorethamine [26], however it is unclear if in vitro radio-sensitization can be compared to the in vivo case.

There have been several modeling efforts in the literature to estimate radiogenic cancer risks [20,22,23], however, there is a lack of mathematical models that estimate both chemotherapy and radiotherapy-induced cancer risks in a concurrent setting. Assuming the dose-response curve as a bell-shaped curve, recent investigations suggest that at high doses of ionizing radiation, cellular repopulation of initiated cells dominates cell killing, due to which the radiogenic risk increases. Epidemiological studies have also suggested that therapy-induced cancer risks decrease at higher doses of ionizing radiation for thyroid tissue. The fact that the dose-response curve takes a bell shape conveys that cell kill dominates the repopulation mechanism in thyroid tissue.

To the knowledge of the authors, this is the first attempt to integrate radiotherapy and chemotherapy protocols along with their interactions, using a biologically motivated mathematical formalism, to estimate concurrent therapy-induced secondary cancer risks. However, the uncertainties that stem from either the lack or quality of input data are considerable. The derivation of the mutation induction rate for breast cancer is, for example, also dependent on the radiation dose to the ovaries and the hormonal status after therapy, but these aspects could not be included in the model due to lack of data. Similarly, the cell kill due to chemotherapy in normal tissues has been fixed to a value close to radiotherapy (α_C_ = 0.2), as literature values were not available. To investigate the robustness of this assumption, we varied the value from 0.1 to 0.3 to assess the change in the parameter we fitted. Additionally, we used the mechlorethamine dose per MOPP cycle as placeholder for the combination of drugs that is used in a different chemotherapy regimen. Therefore, the resulting chemotherapy mutation induction rate γ_C_ per cycle (Table 4) should be taken as order of magnitude estimations for the average combination of drugs used, rather than exact values. In spite of these difficulties, the resulting model can be used to estimate relative differences between competing chemo-radiation schedules.

Another difference between the patients in the studies to contemporary patients is the treatment regimen itself. MOPP was a very popular treatment during the time the patients in these studies [20,22,23] were treated, but has since been superseded by other regimen for many applications. However, secondary cancer induction data for newer regimens, such as ABVE, ABVE-PC, BEACOPP, and others will not be available for many years. Due to the lack of data, we were not able to validate our predictions on an independent dataset, as we extracted the parameter values from the only dataset available that is sufficiently powered.

Radiation therapy induces systemic effects within and outside the treatment field. Some of the indirect effects of radiation include by-stander effects, changes in the immune response, abscopal effects, and others. The biological mechanisms of the aforementioned effects are not yet completely understood. Moreover, the biological and physical principles underlying the synergy between radiation-induced systemic effect and chemotherapy are subject of intensive research efforts.

We hope that our unified chemo-radiation mathematical framework coupled with treatment interaction can be used as a pre-clinical assessment to investigate the impact of concurrent therapy regimen on secondary cancer risks in various primary cancer patients. The same mathematical framework can be applied to other cancer sites as well, however the parameter set for radiation will change and the results will depend on the neighboring organs of the primary cancer. For example, secondary cancer sites for Head and Neck cancer patients include lung and esophagus. The radiobiological parameters for these organs are different compared to the organs of interest in the HL patients. Therefore, the solution space depends on the parameters used in the mathematical framework.

An important clinical application of this formalism is to investigate the temporal administration of concurrent therapy in childhood cancers. As survival continues to rise and early toxicities decrease, the risk of secondary neoplasms will gain weight in the treatment decision process. The presented unified framework can be applied to any concomitant medication administered along with radiotherapy, under the assumption that the dose-response curve can be described by the formalism. This study enhances the understanding of secondary malignancy induction due to concurrent therapy protocols, and could serve as a tool to design efficient therapeutic strategies for childhood cancers that minimize secondary cancer induction.

## 4. Materials and Methods

### 4.1. Definition of Risk

There are several types of therapy-induced secondary malignancy risk parameters defined in the literature, such as life time risk, absolute risk, relative risk, excess relative risk. In this study, we use ERR as a metric to quantify therapy-induced secondary cancer risks. Therapy-induced excess secondary cancer risks are defined as:

(1) ERR=f(D)g(a,e,s,h)

where f(D) is a nonlinear function of the prescribed dose [27]. The output of this function is f(D)=M, which denotes the number of premalignant cells at the end of the treatment. The function g(a,e,s,h) is a nonlinear function that depends on several variables such as gender (*s*), attained age (*a*), exposure age (*e*) and other demographic factors (*h*). In our model, this function is the proportionality factor g that represents all the biological mechanisms that occur during the latency time. Additionally, according to the standard definition, relative risk is defined as RR=1+ERR. Clinically, it is known that relative risks change over time post treatment, however in this investigation we focus on the lifetime risk at the end of treatment. 

### 4.2. Mathematical Framework

In the current study, the initiation–inactivation–repopulation formalism developed previously [19,28,29,30,31] is used to estimate secondary cancer risks, which leads to a bell-shaped dose-response model. This framework incorporates three biological phenomena, namely cell kill effects, repopulation effects and mutation induction. The fundamental rationale behind this model is that it tracks the number of therapy-induced premalignant cells throughout the course of treatment. The assumptions in this framework are the following:

- Radiation and chemotherapy induce cell kill in all compartments, i.e., of normal cells and therapy-induced pre-malignant cells
- Treatment regimen induces normal cells to become pre-malignant
- Mutation induction rate is a function of treatment dose (either chemo or radiation)
- Repopulation of normal and therapy-induced pre-malignant cells occur when treatment starts and continues between fractions and further past the end of the treatment
- Homeostasis regulation of normal tissue is considered
- Age at diagnosis, exposure age and gender are normalized in the ERR function (see Equation (1))
- Radiation is administered using 2 Gy daily with 5 fractions/week with no treatment on weekends [19]
- Combined action of radiation and chemotherapy is incorporated into the formalism by an interacting factor that is initially assumed to be zero.

The function f(D) in Equation (1) represents this initiation–inactivation–proliferation model. Suppose *n*(*t*) and *m*(*t*) denote the number of normal and therapy-induced premalignant cells at time *t*. Then the continuous version of the discrete model developed by Sachs and Brenner [19] can be stated as

(2) $$\begin{array}{l} \frac{dn}{dt}=F(n)-G(n)-R(n)\\ \frac{dm}{dt}=F(m)-G(m)+R(m) \end{array}$$

The functions F,G,R in the above coupled equations represent cell growth (*F*), cell kill (*G*) and mutation induction (*R*) due to therapy. The mathematical model assumes the growth function to be logistic, due to homeostatic regulation of healthy tissues. Therefore, the functions $F(n)=\lambda n\left(1-\frac{n}{N}\right)$ and $F(m)=r\lambda m\left(1-\frac{n}{N}\right),r\lambda >0$ represent the repopulation effect of normal and premalignant cells respectively, during the fractionated treatment with *N* being the initial number of cells and λ denoting the proliferation rate. The factor *r* represents the relative growth of premalignant cells relative to the normal population.

The function $G_{R}(n)=\alpha_{R}d_{R}\sum_{i=1}^{K_{R}}f(t-t_{i})n$ denotes the cell kill effect due to radiotherapy treatment administered over $K_{R}$ fractions, each with a dose $d_{R}$(*Gy*) to the organ at risk. The function *f*(*t*) represents a step function and the irradiation time is approximately τ (minutes) with a dose rate given by d/τ.

In a similar manner, chemotherapy-induced cell kill is given by the function $G_{C}(n)=\alpha_{C}d_{C}\sum_{i=1}^{K_{C}}f(t)\exp(-\alpha_{d}t)n$, where *f*(*t*) represents a step function. There is an exponential decay term within $G_{C}(n)$ that denotes the biological decay rate of the chemotherapeutic agent. The decay rate represents an average quantity, which is a combination of several biological mechanisms such as metabolization, excretion and inactivation.

Finally, mutation induction by ionizing radiation and chemotherapy are given by the functions $R_{R}(n)=\gamma_{R}d_{R}\sum_{i=1}^{K_{R}}f(t-t_{i})n$ and $R_{C}(n)=\gamma_{C}d_{C}\sum_{i=1}^{K_{C}}f(t-t_{i})n$. Here, *γ* describes the mutation induction rate.

So, taking together all the effects described above, the coupled differential equations for radiotherapy are given by:

(3) $$\begin{array}{l} \frac{dn}{dt}=\lambda n\left(1-\frac{n}{N}\right)-\alpha_{R}d_{R}\sum_{i=1}^{K_{R}}f(t-t_{i})n-\gamma_{R}d_{R}\sum_{i=1}^{K_{R}}f(t-t_{i})n\\ \frac{dm}{dt}=r\lambda n\left(1-\frac{n}{N}\right)-\alpha_{R}d_{R}\sum_{i=1}^{K_{R}}f(t-t_{i})m+\gamma_{R}d_{R}\sum_{i=1}^{K_{R}}f(t-t_{i})n \end{array}$$

and for chemotherapy accordingly:

(4) $$\begin{array}{l} \frac{dn}{dt}=\lambda n\left(1-\frac{n}{N}\right)-\alpha_{C}d_{C}\sum_{i=1}^{K_{C}}f(t-t_{i})\exp(-\alpha_{d}t)n-\gamma_{C}d_{C}\sum_{i=1}^{K_{C}}f(t-t_{i})n\\ \frac{dm}{dt}=r\lambda n\left(1-\frac{n}{N}\right)-\alpha_{C}d_{C}\sum_{i=1}^{K_{C}}f(t-t_{i})\exp(-\alpha_{d}t)m+\gamma_{C}d_{C}\sum_{i=1}^{K_{C}}f(t-t_{i})n \end{array}$$

### 4.3. Interacting Term

This factor integrates the interaction of radiation and chemotherapy into the framework as follows:

(5) $$\begin{array}{l} \frac{dn}{dt}=F(n)-G(n)-R(n)-I(n)\\ \frac{dm}{dt}=F(m)-G(m)-R(m)-I(m) \end{array}$$

Many cytotoxic chemotherapeutic agents have radio-sensitizing properties as well. For example, Cisplatin and Temozolomide (TMZ) are administered concurrently with radiotherapy to treat head and neck cancer and Glioblastoma respectively. Several authors have analyzed the interaction of radiation and chemotherapeutic agents in vitro, and have shown an increase in cell kill rate during the small time interval of irradiation (for example, see [32]). However, the complex biological mechanisms of interaction between radiation and various drugs are still unclear. Also, the scarceness of experimental data to understand and quantify the interaction in a concurrent regimen setting makes it complex to derive an exact interacting function between ionizing radiation and chemotherapy. We define the interacting term as a factor that enhances the cell kill mechanism, due to the effect of chemo-radiation given by I(n)=εn and I(m)=εm, where *ε* is the extra cell kill induced by chemo-radiation. It should be noted that this interacting factor is active only during administration of radiation therapy in a concurrent therapy regimen. These assumptions lead to the following formalism:

(6) $$\begin{array}{l} \frac{dn}{dt}=\lambda n\left(1-\frac{n}{N}\right)-\alpha_{R}d\sum_{i=1}^{K_{R}}f(t-t_{i})n-\gamma_{R}d_{R}\sum_{i=1}^{K_{R}}f(t-t_{i})n-\alpha_{C}d_{C}\sum_{i=1}^{K_{C}}f(t-t_{i})\exp(-\alpha_{d}t)n\\ -\gamma_{C}d_{C}\sum_{i=1}^{K_{C}}f(t-t_{i})n-\varepsilon n\\ \frac{dm}{dt}=r\lambda n\left(1-\frac{n}{N}\right)-\alpha_{R}d\sum_{i=1}^{K_{R}}f(t-t_{i})m+\gamma_{R}d_{R}\sum_{i=1}^{K_{R}}f(t-t_{i})n-\alpha_{C}d_{C}\sum_{i=1}^{K_{C}}f(t-t_{i})\exp(-\alpha_{d}t)n\\ -\gamma_{C}d_{C}\sum_{i=1}^{K_{C}}f(t-t_{i})n-\varepsilon m \end{array}$$

Table 9 summarizes the radiotherapy and chemotherapy parameters and their interpretations used in this formalism.

**Figure 2:**
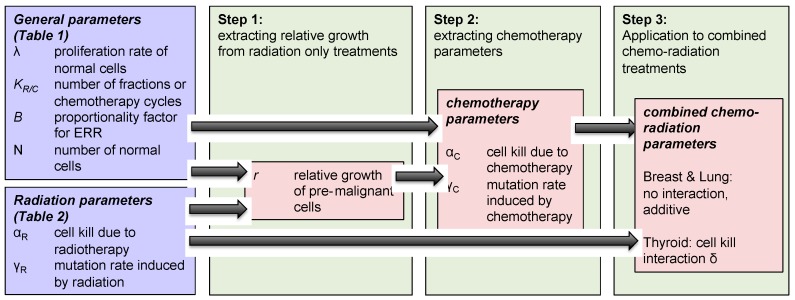
Schematic diagram of the mathematical model formulation to obtain organ-specific synergistic interactions between chemo and radiation.

The methodology used to investigate concurrent therapy secondary cancer risks is described below and illustrated in Figure 2 as well.

- Step 1: We modeled the effect of “radiotherapy only” on secondary cancer risks, and extracted the relative growth rate of radiation-induced pre-malignant cells, using Equation (3) and parameters taken from Table 1.
- Step 2: The same modeled was then applied to analyze the impact of “chemotherapy only” on breast, lung and thyroid secondary cancer risks, using Equation (4) and parameters taken from Table 2. Growth rates of normal and pre-malignant cells used in the radiation model were also used in chemotherapy framework. Using this framework, we extracted the chemotherapy-induced mutation rate parameter for the three organs of interest: breast, lung and thyroid.
- Step 3: Using Equation (6), we investigated the effect of concurrent therapy on secondary cancer risks, and explored if there exists any organ-specific interaction between chemotherapy and radiotherapy with respect to secondary cancer induction.

### 4.4. Epidemiological Data

The parameters in this study are derived from the population-based cohorts taken from Hodgkin’s Lymphoma cancer survivors for breast and lung organs, and childhood cancer survivors’ data for thyroid tissue. The childhood cancer survivors study (CCSS) includes cohorts of childhood cancer survivors diagnosed during the period of 1975–1994 [20,22,23], who survived at least 5 years post treatment. All the survivors from this cohort study were primarily diagnosed with leukemia, Hodgkin’s Lymphoma, non-Hodgkin’s Lymphoma, Wilms tumor, central nervous system tumors, neuroblastoma and bone cancer. The treatment protocol for these patients was radiotherapy, chemotherapy or a combination of radiation and chemotherapy. In total, a population-based cohort of 14,363 [20], 3817 [22], 19,046 [23] patients was considered to analyze and report quantitative associations between radiation, chemotherapy and the corresponding secondary thyroid, breast and lung malignancy risks [20,22,23]. In this study, we investigated if there is any interaction between chemotherapy and ionizing radiation by fitting our mathematical model to the historical data for various critical organs.

## 5. Conclusions

The generalized mathematical framework developed in this study can consider various radiation fractionations as well as different types of chemotherapy regimens. For this purpose, we have used population average parameters derived from the available Childhood Cancer Survivor Study (CCSS) dataset. We showed that the model provides reasonable fits to the patient data and, moreover, that it is able to predict carcinogenic risks. The relevance of our in-silico risk predictions demonstrates the need to consider organ-specific treatment interactions when designing chemo-radiation regimens in the clinical practice to treat primary tumors. Our study can be considered as a pre-clinical assessment of organ-specific treatment interactions and needs further validations using larger secondary cancer data sets, when available in the future. In addition, we anticipate and hope that organ-specific toxicities are also considered when designing clinical trials, involving concurrent regimens.

## Figures and Tables

**Table 1:** Summary of model parameters to estimate radiotherapy-induced secondary cancer risks for breast, lung and thyroid organs.

Parameters	Value	Reference
$K_{R}$	20	[19]
λ	0.4	[19]
N	10^6^	[19]
$\gamma_{R}$	10^−6^	[19]
$r_{breast}$	0.76	[19]
$r_{lung}$	0.96	[19]
$r_{thyroid}$	0.68	Extracted
$g_{breast}$	1.2	[19]
$g_{lung}$	0.18	[19]
$g_{thyroid}$	1.0	Assumption
$\alpha_{Rbreast}$	0.18	[19]
$\alpha_{Rlung}$	0.18	[19]
$\alpha_{Rthyroid}$	0.25	[21]

**Table 2:** Summary of chemotherapy model parameters (taken from literature) and extracted values to estimate chemotherapy-induced secondary cancer risks.

Parameters	Value	Reference
$d_{c}$(Dose per cycle, mg/m^2^)	12	[20,22,23]
Number of chemotherapy cycles	3, 6, 10	[20,22,23]
Average number of days per chemotherapy cycle	30	[20,22,23]
λ	0.4	[19]
$r_{breast}$	0.76	[19]
$r_{thyroid}$	0.68	Extracted
$r_{lung}$	0.96	[19]
$\alpha_{d}$	0.1333	[24]
$\begin{array}{c} \alpha_{Cbreast}\\ \alpha_{Clung}\\ \alpha_{Cthyroid} \end{array}$	0.2	Assumption
$g_{breast}$	1.2	[19]
$g_{thyroid}$	1.0	Assumption
$g_{lung}$	0.18	[19]

**Table 3:** Extracted chemotherapy-induced mutation induction parameter for breast, lung and thyroid tissues.

Organ	Mutation Parameter
$\gamma_{c}$ (breast)	10^−9^
$\gamma_{c}$ (lung)	0.5 × 10^−6^
$\gamma_{c}$ (thyroid)	0.9 × 10^−7^

**Table 4:** Chemotherapy-induced relative risks in breast tissue.

Number of Cycles	Relative Risk (Breast)	
	Model	Data [22]
3	1.0	0.7 (0.3–1.7)
6	1.0	0.6 (0.3–1.1)
10	1.0	0.2 (0.1–1.0)

**Table 5:** Chemotherapy-induced relative risks in lung tissue.

Number of Cycles	Relative Risk (Lung)	
	Model	Data [23]
3	4.1	4.0 (1.3–12.5)
6	7.0	6.2 (2.6–17.1)
10	10.6	13.0 (4.3–45)

**Table 6:** Chemotherapy-induced relative risks in thyroid tissue.

Number of Cycles	Relative Risk (Thyroid)	
	Model	Data [20]
5	4.61	1.8 (0.3–10.0)
10	6.02	9.4 (1.4–56.8)

**Table 7:** Concurrent therapy: Relative risks of secondary breast, lung and thyroid cancers predicted by a mathematical model vs. Historical data [20,22,23].

Organ	Relative Risks	
	Model	Data [20,22,23]
Breast	9.37	1.4 (0.5–4.2)
Lung	9.26	8.0 (3.6–18.5)
Thyroid (low/medium)	7.73	2.3 (1.3–4.5)
Thyroid (high)	7.24	2.8 (1.1–6.7)

**Table 8:** Concurrent therapy: relative risk of secondary thyroid and breast cancers predicted by mathematical model vs. historical data [20,22] with the chemo-radiation interacting factor.

Organ	Relative Risks	
	Model	Data [20,22]
Thyroid (low/medium)	4.56	2.3 (1.3–4.5)
Thyroid (high)	6.00	2.8 (1.1–6.7)
Breast	3.20	1.4 (0.5–4.2)

**Table 9:** Description of model parameters for the mathematical framework.

Parameters	Interpretation
$K_{R}$	Number of fractions
$K_{C}$	Number of chemotherapy cycles
$d_{R}$ (Gy)	Radiation dose per fraction
$d_{C}$ (mg/m^2^)	Chemotherapy dose per cycle
λ (per day)	Proliferation rate of normal cells
r	Relative growth of premalignant cells
rλ	Proliferation rate of premalignant cells
$\gamma_{R}$ (per Gy)	Mutation rate induced by radiation
$\gamma_{C}$ (per chemotherapy dose)	Mutation rate induced by chemotherapy
$\alpha_{R}$ (per Gy)	Cell kill due to radiotherapy
$\alpha_{d}$ (per unit time)	Decay rate of chemotherapy drug
$\alpha_{C}$ (per chemotherapy dose)	Cell kill due to chemotherapy
